# Pharmacokinetics of immunosuppressive agents during hemoperfusion in a sheep model

**DOI:** 10.3389/fmed.2023.1258661

**Published:** 2023-10-20

**Authors:** Bettina Leber, Uwe Liebchen, Lisa Rohrhofer, Jennifer Weber, Teresa Klaus, Joerg Scheier, Robert Sucher, Philipp Stiegler

**Affiliations:** ^1^General, Visceral and Transplant Surgery, Department of Surgery, Medical University of Graz, Graz, Austria; ^2^Department of Anesthesiology, LMU Hospital, Munich, Germany; ^3^CytoSorbents Europe GmbH, Berlin, Germany

**Keywords:** CytoSorb, hemoadsorption, hemoperfusion, immunosuppressants, immunosuppressant drugs, adsorption, blood purification

## Abstract

**Introduction:**

Hemoadsorption shows promising signals in organ preservation and post lung transplantation. However, its potential impact on the pharmacokinetics of immunosuppressant drugs (ID) is still unknown.

**Methods:**

In this interventional study, CytoSorb® hemoperfusion was tested in healthy sheep (*n* = 5) against a sham extracorporeal circuit (*n* = 3). Seven different ID (tacrolimus (TAC), cyclosporin A (CYA), mycophenolate mofetil (MMF), everolimus (EVER), basiliximab (BAS), methylprednisolone (MP) and prednisolone (PRED)) were administered in clinically relevant doses and combinations. Their levels were measured repeatedly in blood samples from the extracorporeal circulation over 6 h following administration. Population pharmacokinetic modeling analysis (NONMEM® 7.5) was performed.

**Results:**

Negligible clearance was observed for PRED and BAS. For all other substances, a saturable adsorption sub-model with linear decrease of the adsorption effect over the adsorbed amount best described the measured concentrations. The maximum absolute adsorbed amounts (95% CI) for TAC, CYA, MMF, EVER, and MP were 0.040 (0.028–0.053), 1.15 (0.39–1.91), 4.17 (2.00–6.35), 0.0163 (0.007–0.026), and 53.4 mg (20.9–85.9), respectively, indicating an adsorption of less than 5% of the daily administered dosages for all investigated substances.

**Discussion:**

In this large animal model, CytoSorb® hemoperfusion appears to have a limited effect on the clearance of tested ID.

## Introduction

Immunosuppressant drugs (ID) are used for various indications. Beyond their long-term use in an increasing number of patients for a range of conditions (1), ID are of vital importance for over 120,000 patients who annually undergo solid organ transplantations worldwide (2). However, scarcity of organs presents the most challenging barrier with long waiting lists and many patients failing to receive a transplant in time (3).

In this context, *ex-vivo* lung perfusion (EVLP) has recently gained a role in donor lung assessment, but also donor lung preservation, with the target to increase the donor pool and improve organ quality. However, cytokine accumulation during EVLP has been shown to correlate with worse outcomes after lung transplantation (4). Consequently, cytokine adsorption has recently been evaluated in this setting (5).

The CytoSorb® hemoadsorption device (CytoSorbents Corporation, NJ, USA), has been marketed and licensed for extracorporeal cytokine removal since 2011 within the European Union (6). It can be integrated into continuous renal replacement therapy (CRRT) or hemoperfusion circuits as well as into bypass circuits within extracorporeal membrane oxygenation (ECMO) machines or cardiopulmonary bypass (CPB). The cartridges contain highly biocompatible polystyrene divinylbenzene copolymer beads, coated with polyvinylpyrrolidone capable of removing small to midsize hydrophobic molecules up to a molecular weight of approximately 60 kDa (7) in a concentration-dependent manner. Various terms are used to describe the use of hemoadsorption techniques. In the following article, we will speak of “hemoperfusion” if the adsorption device is used in a stand-alone set-up and of “hemoadsorption” if use is in conjunction with other procedures like concomitant renal replacement therapy or when referring to literature.

Along with its main purpose of reducing excessive levels of cytokines, bilirubin (8), and myoglobin (9), hemoadsorption has also been shown to adsorb certain drugs from the blood (10). Although this can be of therapeutic advantage in the case of intoxication (11) or the prevention of bleeding by removal of antithrombotic agents (12), it may also imply a significant risk for the patient when unwanted removal of potentially life-saving medication occurs. Regarding anti-infectives, a standardized animal model in pigs showed an existing but overall limited effect on the pharmacokinetics of the majority of examined drugs (13).

Knowledge about the effect of hemoadsorption on the pharmacokinetics of immunosuppressants, however, remains limited. So far, post market surveillance has not suggested major adverse events with the use of CytoSorb® in patients on immunosuppression. To our knowledge, immunosuppressant drug levels have never been systematically investigated in blood taken directly from the inlet and outlet of the cartridge *in-vivo*. Published literature consists mainly of single cases with a focus on clinical outcome and if any, only on performing measurements of systemic drug levels (14–17). In summary, these reports do not represent sufficient safety information and lack consistency and reproducibility.

To address the urgent need for more reliable data we designed an experimental animal study to investigate the potential impact of hemoperfusion on the pharmacokinetics of immunosuppressants to generate standardized data contributing to the safety portfolio of the device.

## Materials and methods

### Animals

Fifteen 5-year-old ewes with a body weight of approximately 85 kg, purchased from a legal breeder (Christoph Priedl, Austria; LFBIS-/AMA-KI.Nr: 3335976), were housed at the Institute for Biomedical Research (Medical University of Graz, Graz, Austria) and were taken to the pasture daily. The study protocol was approved by the Austrian Committee for Animal Trials (Approval No: 2020-0.437.202). Animals had free access to fresh hay or grass and water as required. The present report was prepared following the ARRIVE 2.0 guidelines by the NC3R^s^ (National Centre for the Replacement Refinement and Reduction of Animals in Research).

### Catheter implantation and extracorporeal circulation

The dialysis catheter was implanted according to standard procedures. After appropriate shaving and disinfection, the right jugular vein was punctured under general anesthesia and a 60 cm central venous catheter (Palindrom; Medtronic) was placed according to the Seldinger technique. Blood aspiration and flushing of the catheter with heparinized saline were performed to verify the correct position. The jugular catheter was then sutured to the skin, the site covered with a disinfectant swab, and the catheter secured to the neck with a bandage.

The central venous access was secured to the animal in such a way that the sheep could not remove it during normal movement. Daily catheter care consisted of inspection and cleaning of the entry site (bandage change if necessary), flushing of the catheter with heparinized saline solution, and blocking of the catheter lumen with Taurolock® Hep500. If the central venous access was no longer useable, a new catheter was placed in the jugular vein on the contralateral side in the same way as described above.

**Figure 1:**
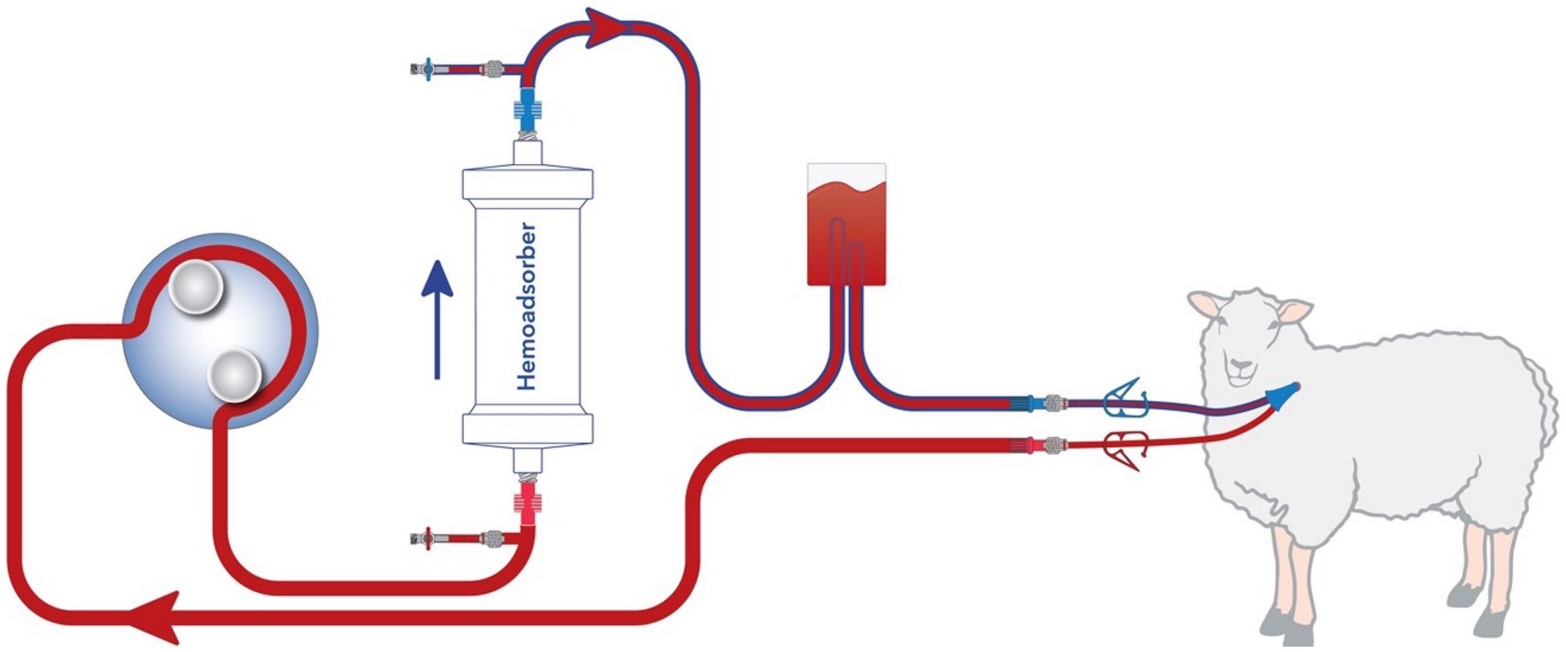
Extracorporeal circulation with hemoadsorber.

A schematic representation of the extracorporeal circuit (ECC) is shown in Figure 1. It was established in all animals with a dedicated device [BM11a, Baxter, Deerfield, IL, USA) and corresponding circuit (BM11-Lines-BA-HP tube system set, Baxter, Deerfield, IL, USA]. For animals allocated to the intervention group, a CytoSorb® cartridge was inserted in the ECC. For those allocated to the control group, no cartridge was integrated (sham hemoperfusion). The blood flow rate was kept at around 120 mL/min throughout the experiment. Immediately prior to the start of hemoperfusion or sham procedure animals were anticoagulated by intravenous application of 10,000 IU of Heparin. To maintain anti-coagulation 5,000 IU heparin was administered hourly to the inlet port.

### Drug administration

**Figure 2:**
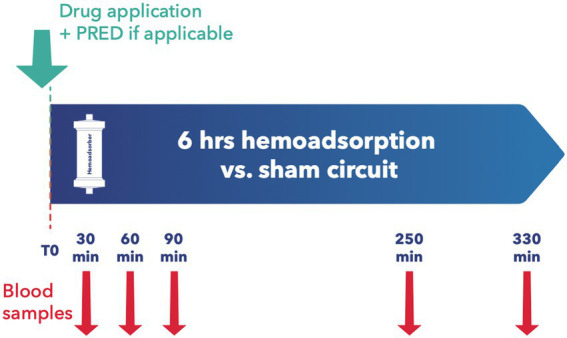
Experimental design for groups 1–4. No control group for group 4 (basiliximab).

**Figure 3:**
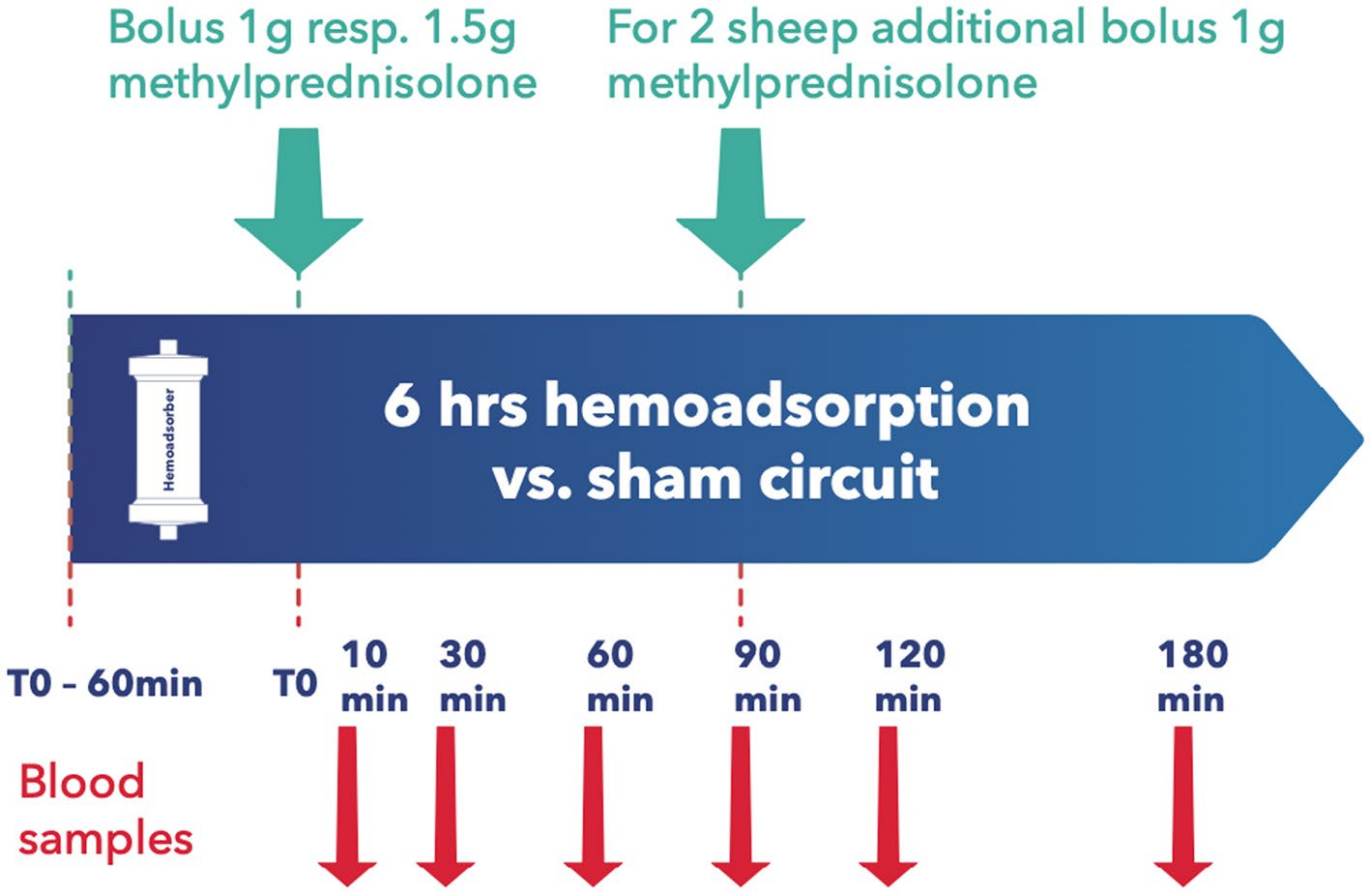
Experimental design for methylprednisolone.

All animals were granted a 14-day acclimatization period prior to experiment onset. After insertion of a central venous catheter as described below, the desired medication was applied, with subsequent random assignment to the intervention or control group. Since most immunosuppressants are absorbed enterally, the absorption behavior of Cyclosporin A (CYA) Tacrolimus (TAC), Mycophenolat Mofetil (MMF), and Everolimus (EVER), was tested in 4 pilot animals and data included for analysis. Since EVER is only available as an oral preparation and TAC could not be administered continuously via syringe pump over 24 h due to the trial set-up and limited staffing, all tested ID except MP and BAS were administered orally. Drugs were administered to the animals twice daily, as described elsewhere (18, 19). For TAC, EVER, and CYA, blood levels were determined three times weekly and if necessary, the doses were adapted accordingly. Upon achieving target blood levels as defined by current recommendations (20–23) an extracorporeal circuit with (intervention group) or without (control group) hemoadsorber was applied for a total duration of 6 h. On the morning of the first post-intervention day, blood levels of the respective immunosuppressive drugs were determined, and the doses were adjusted to keep up the required target levels. PRED was applied for all combination therapies in the morning directly prior to the onset of the extracorporeal intervention. See Table 1 for dosages and group distribution. For each immunosuppressant combination, five animals were allocated to the intervention group and compared to 3 control animals. BAS (20 mg) was applied in 3 animals directly before the onset of the extracorporeal intervention. For BAS no control animals were examined as due to its large molecular size of around 144 kDa no elimination by the device was to be suspected (24) (see Figure 2). For Methylprednisolone (MP) two sheep served as control and received 1 g MP without integration of the adsorber. In the study group, two scenarios simulating intraoperative application of the drug during running CPB (mimicking a heart transplant scenario) plus hemoadsorption were tested. We investigated either an increased starting dose (1.5 g MP) or the application of an additional dose of 1 g after 1.5 h (see Figure 3).

**Table 1:** Examined substances and respective dosages.

Substance class	Substance	Dosing	Target level	
Calcineurin inhibitors	Tacrolimus	2×10–12 mg^a^	6–8 ng/mL	
	Ciclosporin A	2×600–1,000 mg^a^	80–100 ng/mL	
Proliferation inhibitors	MMF	2×1 g		
	Everolimus	2×3–8.25 mg^a^	3–8 ng/mL	
Antibodies	Basiliximab	20 mg	–	
Steroids	Prednisolone	10 mg	–	
	Methylprednisolone	2 sheep 2×1 g, 2 sheep 1×1,5 g, 2 sheep (Control) 2×1 g	–	
**Group 1**	**Group 2**	**Group 3**	**Group 4**	**Group 5**
TAC, MMF, PRED	CYA, MMF, PRED	EVER, MMF, PRED	BAS	MP

Substances were applied in clinically relevant combinations as displayed. ^a^Dosages, presented as min – max, were adapted individually according to blood levels of the respective drug.

### Blood samples and laboratory analysis

Baseline blood samples were collected before administration of the immunosuppressants. For combinations of TAC, CYA, MMF, EVER, and PRED blood samples were collected immediately prior to and at 30, 90, 250, and 330 min after the adsorber/sham procedure initiation (see Figure 2). For MP measurement samples were taken at 10, 30, 60, 90, 120, and 180 min after administration of the first MP dosage (see Figure 3). For animals allocated to the intervention group, two samples per time point, before (inlet) and after (outlet) the adsorber, were collected. For those allocated to the control group, we also collected samples from the extracorporeal circuit. Two ethylenediaminetetraacetic acid (EDTA) tubes and one serum tube were collected for inlet and outlet samples, respectively. Serum was separated from cells by centrifugation at 1,900× *g* for 10 min. The serum was split into two 2 mL Eppendorf tubes (Eppendorf AG, Hamburg, Germany) and frozen at −80°C until determination of PRED levels by a commercially available ELISA kit (MyBiosource, San Diego, USA).

In the case of TAC, EVER, and CYA whole blood levels and MMF EDTA plasma levels were measured at the laboratory of University Hospital Graz. TAC, CYA, and EVER levels were determined by LC–MS/MS (QTRAP 4500, Sciex) combined with a Shimadzu HPLC System whereas for detection of MMF levels, an Agilent HPLC System (G1310A) in combination with a multiple wavelength detector (G1365B) was used. For BAS the final absorbance was read at 490 and 630 nm (for correction) on an ELx808 ELISA plate reader (BioTek, VT, USA) at Centre Hospitalier Regional et Universitaire de Tours, France. Methylprednisolone was quantified using a sensitive ultra-performance liquid chromatography–tandem mass spectrometry (UPLC–MS/MS; ThermoFisher Scientific, San Jose, CA, USA) at University Hospital Reims, France.

### Determination of clearance and elimination by the adsorber

In the first step, the course of the drug levels measured pre- and post-adsorber were compared to controls without adsorber (see Supplementary Figures 1–4). For BAS only pre- and post-adsorber levels were compared regarding each time point.

In the second step, clearances were calculated according to the following formula:


$$CL=\frac{Ci-Co}{Ci}\;x\;FL$$

where CL = clearance (in mL/min), Ci = inlet drug concentration, Co = outlet drug concentration, and *FL* = plasma/serum flow through the adsorber (in mL/min), calculated as blood flow×(1-hematocrit). Hematocrit was calculated as 33% according to the standard value in sheep (25). The drug elimination rate (E) was calculated as E = Cl × Ci.

### Pharmacokinetic parameters calculations

In the third step, non-linear mixed effect modeling was performed for all substances with measurable clearance in pre- versus post-adsorber samples using the software NONMEM 7.5 (NONMEM, Version 7.5; Icon Development Solutions, Ellicot City, MD, USA) to investigate adsorption characteristics and provide drug clearance over time. Graphical and statistical analysis was performed using R (Version 4.0.2).^1^

To determine the pharmacokinetics of the administered drugs without adsorption-related effects in sheep, hemoperfusion as a covariate was initially ignored. Based on an initial graphical evaluation of the concentration-time-profile, for orally administered drugs (CYA, MMF, TAC, EVER) only a one-compartment model was investigated, while for MP a one- and a two-compartment model was examined. For the orally administered drugs, volume of distribution (V) and clearance (CL) were estimated in relation to bioavailability (V/F and CL/F). To account for a possible delay to the onset of adsorption, a lag time was evaluated for orally administered drugs (ALAG). Models were selected based on the plausibility and identifiability of the parameters and statistical criteria (∆ Objective function value (OFV) > 3.84 for one additional parameter, Akaike information criterion). Structural parameters were assumed to be log-normally distributed and interindividual variability was evaluated for all parameters. In addition to numeric criteria, the prediction quality of the models for the measured concentrations was evaluated employing goodness of fit plots.

Second, the adsorption characteristics of hemoperfusion were investigated. Therefore, all parameters of step 1 except clearance were fixed and an additional clearance-pathway from the central compartment was added for the cartridge evaluating saturable and non-saturable CL by CytoSorb®, see Eqs 1 and 2. For the saturable CL via a linear decrease model (Eq. 2) the adsorption rate was linked to the maximum CL ( $CL_{\max}$ ), the drug amount already adsorbed at the cartridge ( $A_{Cytosorb}\left(t\right)$ ) and the maximum drug amount that can be adsorbed ( $A_{\max}$ ):

Formula for constant adsorption (k_constant_ = elimination rate constant):


(1) $$CL_{Cytosorb}\left(t\right)=bloodflow.k_{constant}\;\;$$

Formula for saturable adsorption:


(2) $$CL_{Cytosorb}\left(t\right)=CL_{\max}.\left(1-\frac{A_{Cytosorb}\left(t\right)}{A_{\max}}\right)$$

Selection of an appropriate model was based on the precision and plausibility of parameter estimates and statistical criteria (see above).

## Results

First, drug levels measured pre- and post-adsorber were compared to controls without adsorber (see Supplementary Figures 1–4). If direct adsorption (difference pre- and post-measurements) took place it occurred mostly within the first 1–2 h of device exposure. For BAS only pre- and post-adsorber measurements were compared and systemic blood levels showed only a slow decrease over time (see Supplementary Figure 5).

In the second step of analysis, cross-adsorber clearance was calculated from pre- versus post-adsorber measurements and flow rate. Clearance was shown to be negligible both for PRED and BAS (see Supplementary Figures 6–8).

Finally, for all substances that showed relevant clearance rates (TAC, MMF, EVER, CYA, and MP), pharmacokinetic modeling was performed. A one-compartment model with linear elimination and proportional residual variability adequately described the pharmacokinetics of orally administered drugs, while a two-compartment model with linear elimination and a combined proportional and additive residual variability model best-described data of MP. Interindividual Variabilities (IIVs) were included for CL or CL/F, respectively, for all drugs and additionally for V/F for TAC. A lag time improved the model for TAC (15.3 h), MMF (4.7 h), and EVER (4.1 h) and was consequently incorporated into the models. Eta shrinkage was low (<16%) indicating that all individuals contributed to the estimated IIVs. While most parameters could be estimated with high precision (Relative Standard Error (RSE) <30%), few parameters could be identified with only moderate precision (RSE 30–50%, e.g., V/F and first order oral absorption rate (KA) for EVER and CYA), presumably due to the limited number of individuals and the rather sparse sampling schedule during the initial period after oral administration. Residual variabilities were in an acceptable range (16.4–34.5% Coefficient of variation (CV)) for all drugs (see Supplementary Table 1).

**Figure 4:**
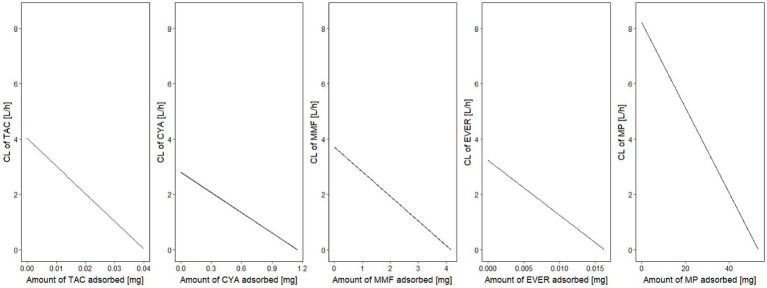
Clearance of the investigated drugs by the hemoadsorber. For all drugs, a linear decrease of clearance indicated saturation of the adsorption kinetics depending on the amount already adsorbed. CL, clearance; TAC, tacrolimus; MMF, mycophenolate mofetil; EVER, everolimus; MP, methylprednisolone.

**Figure 5:**
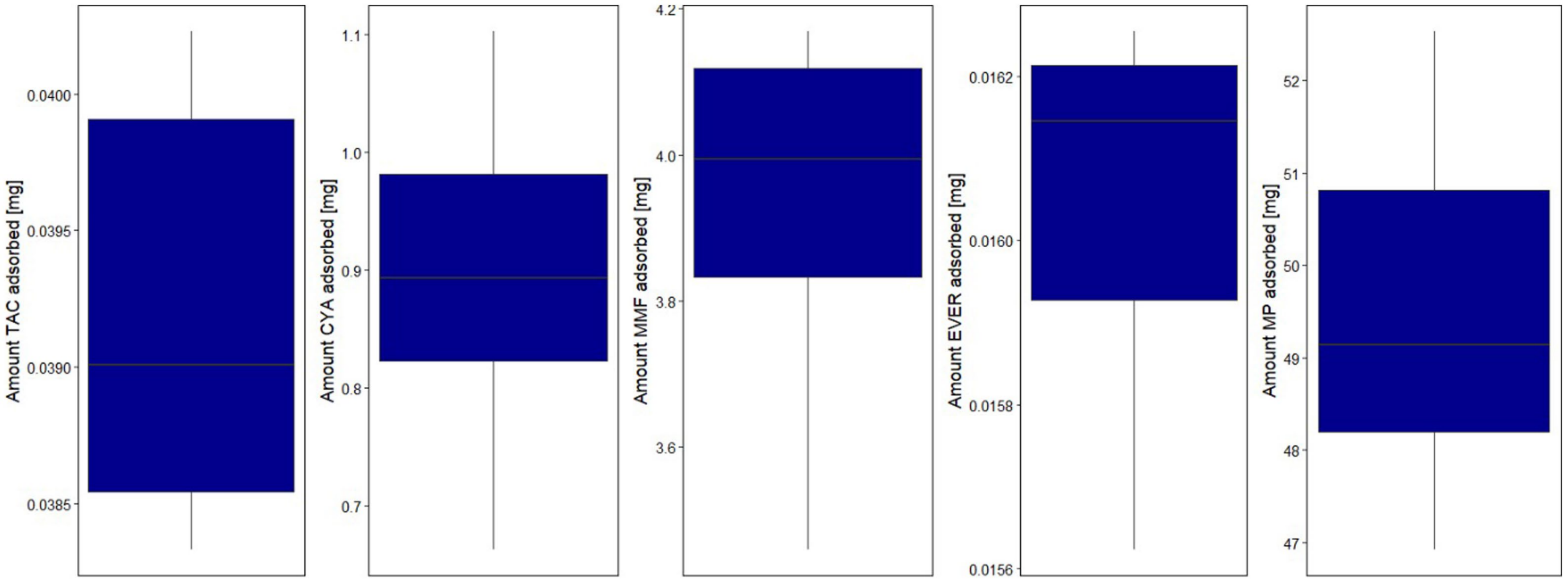
Boxplot of the total estimated adsorbed amount for the investigated drugs by hemoperfusion. Lower and upper box boundaries: 25th and 75th percentiles, respectively; line inside box: median; lower and upper error lines: 10th and 90th percentiles, respectively; TAC, tacrolimus; MMF, mycophenolate mofetil; EVER, everolimus; MP, methylprednisolone.

In the second step of modeling, adsorption by the device was investigated. Evidence of adsorption was noted for all drugs except BAS and PRED, and was therefore included in the models. The (sub-)model representing a linear decrease (Eq. 2) of adsorption rate corresponding to saturable kinetics and characterizing A_max_ and CL_max_ was better (∆OFV: CYA, MMF, TAC, EVER, MP: −13.0, −19.8, −34.4, −16.6, −39.0, *p* < 0.001) than constant adsorption without saturation for all drugs. Estimated maximum adsorbed amounts and CL_max_ of CYA, MMF, TAC, EVER, and MP were 1.15 mg/2.80 L/h, 4.17 mg /3.71 L/h, 0.04 mg /4.02 L/h, 0.0163 mg /3.23 L/h and 53.4 mg /8.21 L/h (see Figures 4, 5). Of note, the estimated CL_max_ of MP was higher than the blood flow, but the confidence interval included the value of 7.2 L/h (95% CI: 6.16–10.25 L/h). For further information see Supplementary Table 2.

A separate residual variability for samples before (=plasma) and after the cartridge was estimated and residual variabilities post adsorber was <30% CV for all drugs (combined residual variability model for MP) indicating adequate representation of the data. Adequate representation of the data was confirmed by graphical evaluation methods stratified by pre- and post-adsorber (see Supplementary Figure 9). After adding the hemoperfusion elimination pathway systemic clearance decreased by less than 10% for all drugs indicating that hemoperfusion elimination accounted for only a small proportion of the total clearance. Overall, the maximum adsorbed amounts indicate an adsorption of less than 5% of the daily administered dosages for all tested substances.

## Discussion

### Key findings

Negligible clearance was observed in the measurements before and after the adsorber for PRED and BAS. For all other substances, a saturable adsorption sub-model with linear decrease of adsorption efficiency over the adsorbed amount best described the results. The maximum absolute adsorption amounts implied an adsorption rate of less than 5% of the daily administered doses for all tested substances.

### Mechanistic considerations for drug removal

**Figure 6:**
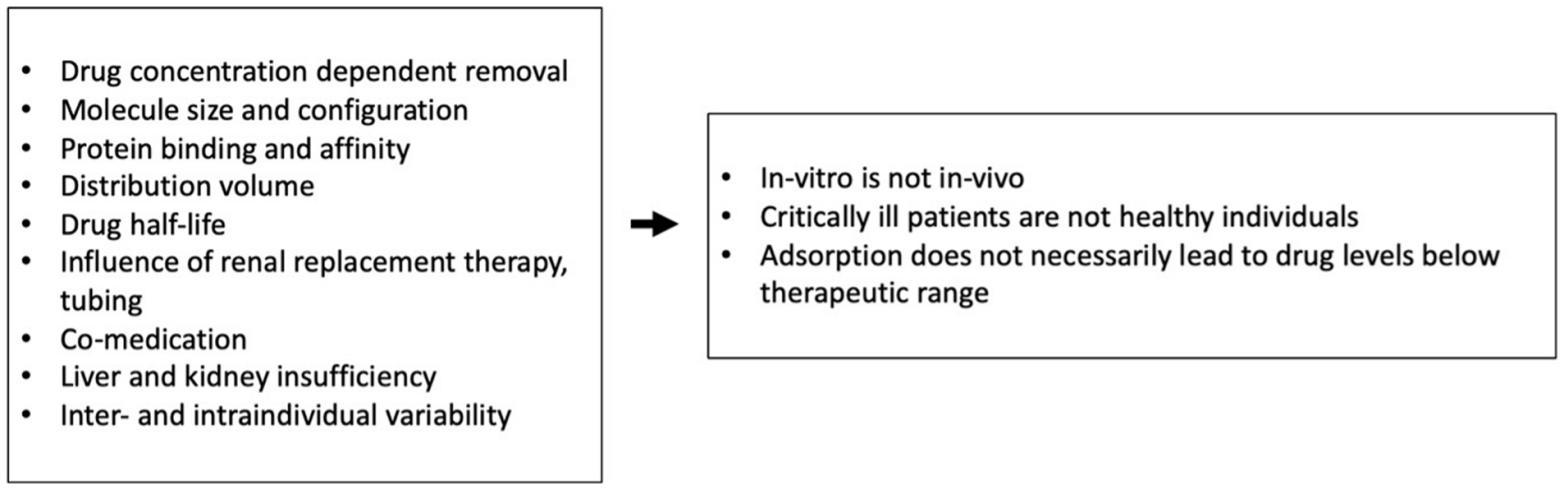
Influencing factors and their consequences on examination of hydrophobic protein-bound substances (8, 11).

The possibility of inadvertent removal of medications in critically ill patients is an important issue that must be considered with the use of all extracorporeal therapies (10, 26). To evaluate the clinical significance of potential drug removal, several factors should be taken into account. These include the patient’s unique medical condition, any other extracorporeal therapies being used concurrently, the length of time the device will be in use, and whether the drug has just been administered or has reached steady-state levels in the patient’s system. Data on the influence of other extracorporeal therapies such as CPB or renal replacement therapy on ID are very scarce and recommendations on necessary dosing adaptations are lacking (27–29). Since hemoadsorption mainly targets hydrophobic substances which are usually protein-bound, other influencing factors such as the distribution volume must be considered when examining and interpreting the impact of such devices on drug levels (Figure 6). Therefore, drug removal data from *in-vitro* experiments are informative but not necessarily directly transferable to more complex *in-vivo* conditions (10).

### Comparison with previous studies

Preexisting data on hemoadsorption in the context of immunosuppressive agents are scarce and partly contradictory. An early *in-vitro* study using uremic blood suggested moderate removal of TAC and CYA (30). Another more recent *in-vitro* study with whole blood as the carrier solution showed, in contrast, insignificant removal of tacrolimus (Hartmann J, 2017, Report P-2017-09 - *In vitro* Adsorption von Immunsuppressiva durch Cytosorb. University of Krems, Austria, data on file). The same investigation found moderate removal of CYA and high removal of mycophenolate and MP. However, as mentioned above such *in-vitro* have several limitations, and their results are not necessarily transferrable to *in-vivo* settings (Figure 6).

Regarding corticosteroids, *ex-vivo* elimination has been shown for methylprednisolone in a lung perfusion model (31) and for cortisol in brain-dead humans (32). In contrast, in an interim analysis in patients with myocardial revascularization divided into three different groups (one of them with a hemoadsorber integrated into the CPB) all hormone levels including cortisol remained comparable between groups suggesting no clinically relevant removal by the device (15).

In general, the use of hemoperfusion in the setting of transplantation is currently a topic of intense investigation which shows the importance of the systematic safety evaluation provided by this study but also the potential impact in clinical outcomes. A recent porcine acute respiratory distress syndrome (ARDS) lung transplantation model showed that the use of the extracorporeal blood purification device CytoSorb® during EVLP and for 12 h post-transplantation was able to restore lung function and reduce primary graft dysfunction (5). The authors suggest that this approach could increase not only the availability of donor organs but also increase tolerability by recipients. In another large animal model, the use of CytoSorb® during EVLP improved airway pressure and dynamic compliance, and reduced electrolyte imbalance and pulmonary edema (4). When lungs were treated with hemoadsorption during EVLP and subsequently transplanted, graft function was better when compared to untreated control lungs (31). In contrast, another recent porcine study investigating the use of hemoadsorption during reperfusion for 6 h in a lung transplant model failed to show similar effects (33).

Two single cases report on good organ function after 1 year post liver transplantation with the perioperative use of MP, BAS, and MMF (34), and 6 months after heart transplantation with the use of TAC, MMF, and PRED (16). Lastly, in an open randomized trial in 30 patients with CytoSorb® versus 30 without, safety interim analysis regarding rejection/mortality showed no harm or disadvantage for any group (Nemeth E, 2019, Reduction of Inflammation in Heart Transplantation, presented at 33^rd^ European Association for Cardiothoracic Surgery – EACTS congress, Lisbon, Portugal). Of note, our data show that most of any adsorption occurs within the first 1–2 h of device exposure which is in line with previous studies (13).

### Implications for clinicians and policymakers

There are various clinical settings in which the use of ID plays a major role, not only during and after transplantation of solid organs, stem cells, or bone marrow, but also in long term treatment of autoimmune diseases. According to our data, we can suggest some initial recommendations when using hemoperfusion in this patient cohort. However, it remains important to conduct human studies before implementing these recommendations into clinical practice.

In this large animal model, hemoperfusion had a very limited effect on the clearance of ID. These findings provide reassuring information for the safe use of the device in patients receiving ID. However, the need for regular blood level checks of immunosuppressants in clinical routine remains unchanged, and therapeutic drug monitoring during hemoadsorption therapy is recommended as with any other extracorporeal circuit. For BAS we were able to exclude any removal in this study which will reassure the usage of the device in patients on other antibody treatments with a similarly high molecular weight.

### Strengths and limitations

This study provides the first standardized data on the possible influence of the hemoadsorption device and also examines an extracorporeal circuit on ID. To bridge the gap between benchtop results and the clinic, *in-vivo* preclinical trials are indispensable given the importance of immunosuppressants and the specific characteristics of the hemoadsorption device. Immunosuppressants have been tested in sheep before (35) and hemoperfusion has been used before in an animal septic shock sheep model (36). In our study sheep were chosen rather than pigs because they could be kept awake without the need for anesthesia for the whole duration of this study. In general, of course, any animal model comes with several limitations that must be considered when transferring the results of experiments to humans (37). Furthermore, the relatively small number of animals may compromise the firmness of conclusions drawn from the study. Also, we tested the substances in healthy sheep without allowing for influences on pharmacokinetics and dynamics resulting from pathophysiological changes seen in the critically ill. All tested ID except MP were administered orally which will presumably show different bioavailability compared to humans. To counteract these limitations, we measured blood drug levels directly within the extracorporeal circuit to assess direct removal from the blood. To gain detailed insights into elimination processes after oral administration, pharmacokinetic compartmental models were developed representing non-linear processes like adsorption more accurately compared to non-compartmental methods. Lastly, substances were administered in combinations which might have a reciprocal influence on adsorption. However, these groups were designed in line with clinical practice and therefore reflect the interactions expected in daily routine (38, 39).

## Conclusion

Herein we report the first standardized examination of the possible influence on ID blood levels for a hemoadsorption device, as well as the examination of the impact of any extracorporeal circulation on ID. We were able to show a lack of removal for basiliximab and prednisolone and very limited influence on the blood levels of tacrolimus, cyclosporin A, mofetil mycophenolate, everolimus, and methylprednisolone. PK modeling revealed negligible clearance attributable to hemoadsorption for the latter substances. Despite the described limitations of the animal model, this represents important safety information for the usage of the CytoSorb® device in patients on immunosuppressants, both in the context of organ transplantation and long-term treatment for other reasons. Clinical decision-making should always be supported by therapeutic drug monitoring whenever available. Ideally, clinical studies in patients should be conducted to confirm our findings.

## Data availability statement

The raw data supporting the conclusions of this article will be made available by the authors, without undue reservation.

## Ethics statement

The animal study was approved by Austrian Committee for Animal Trials, BMBWF – V/3b, Minoritenplatz 5, 1010 Wien, Austria. The study was conducted in accordance with the local legislation and institutional requirements.

## Author contributions

BL: Investigation, Writing – review & editing, Conceptualization, Data curation, Formal analysis, Methodology, Visualization, Writing – original draft. UL: Methodology, Visualization, Writing – original draft, Software, Writing – review & editing, Formal analysis. LR: Investigation, Writing – review & editing. JW: Investigation, Writing – review & editing. TK: Conceptualization, Project administration, Writing – original draft, Writing – review & editing. JS: Conceptualization, Methodology, Supervision, Writing – review & editing. RS: Investigation, Writing – review & editing, Supervision. PS: Conceptualization, Investigation, Methodology, Writing – review & editing, Supervision.

## Figures and Tables

**Table:** 

A_half_	Drug amount associated with half of the maximum adsorption capacity
ALAG	Delay of oral adsorption after oral intake
A_max_	Maximum drug amount that can be adsorbed
ARDS	Acute respiratory distress syndrome
BAS	Basiliximab
CA	California
Ci	Inlet drug concentration
CL	Clearance
Co	Outlet drug concentration
CL_max_	Maximum clearance
CPB	Cardiopulmonary bypass
CRRT	Continuous renal replacement therapy
CV	Coefficient of variation
CYA	Cyclosporin A
DAMPS	Damage associated molecular patterns
E	Elimination rate
ECC	Extracorporeal circulation
ECMO	Extracorporeal membrane oxygenation
EDTA	Ethylenediaminetetraacetic acid
ELISA	Enzyme-linked immunosorbent assay
Eq.	Equation
EVER	Everolimus
EVLP	*Ex-vivo* lung perfusion
F	Bioavailability
FL	Plasma/Serum flow through the adsorber
HPLC	high performance liquid chromatography
ID	Immunosuppressant drugs
IIVs	Interindividual Variabilities
KA	First order oral absorption rate
LC–MS/MS	Liquid Chromatography with tandem mass spectrometry
MMF	Mycophenolat Mofetil
MP	Methylprednisolone
OFV	Objective function value
PAMPS	Pathogen associated molecular patterns
PK	Pharmacokinetics
PRED	Prednisolone
Q	Intercompartmental clearance
RSE	Relative standard error
TAC	Tacrolimus
USA	United States of America
V	Volume of distribution
V1	Central volume of distribution
V2	Peripheral volume of distribution

## Footnotes

^1^CRAN.R-project.org
