# Supplementary material for: Pharmacokinetics of immunosuppressive agents during hemoperfusion in a sheep model
Source: Front Med (Lausanne). 2023 Oct 20;10:1258661. doi: 10.3389/fmed.2023.1258661 (PMC10623319; doi:10.3389/fmed.2023.1258661)
Supplement: Supplementary file 1 [file Data_Sheet_1.DOCX]

# Supplemental digital content


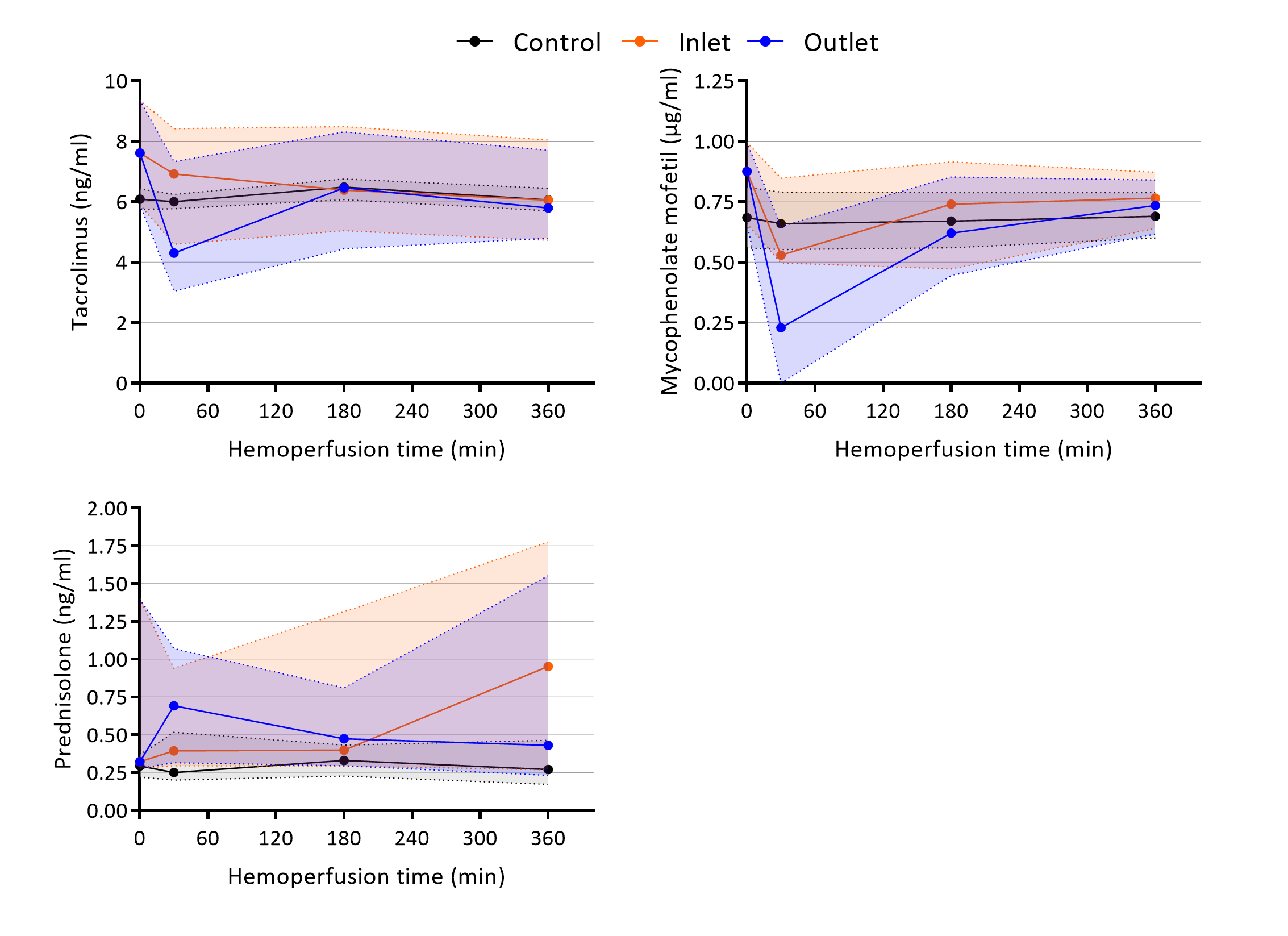


Supplemental Figure 1. Tacrolimus, Mycophenolate Mofetil and Prednisolone: Drug levels measured at the In- and Outlet of CytoSorb® versus controls. Data are displayed as median (line) and interquartile range (shaded area).


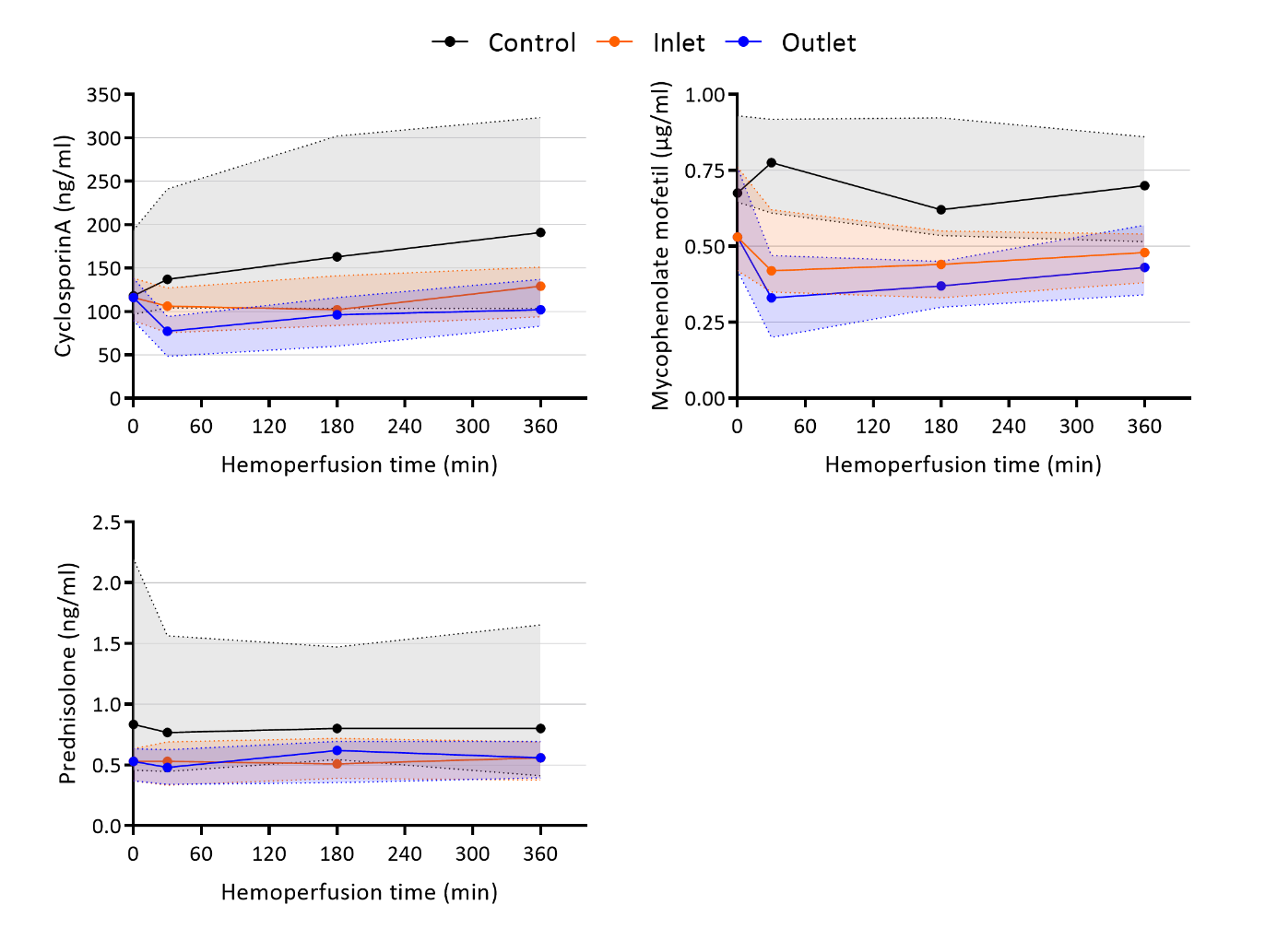


Supplemental Figure 2. Cyclosporin A, Mycophenolate Mofetil and Prednisolone: Drug levels measured at the In- and Outlet of CytoSorb® versus controls. Data are displayed as median (line) and interquartile range (shaded area).


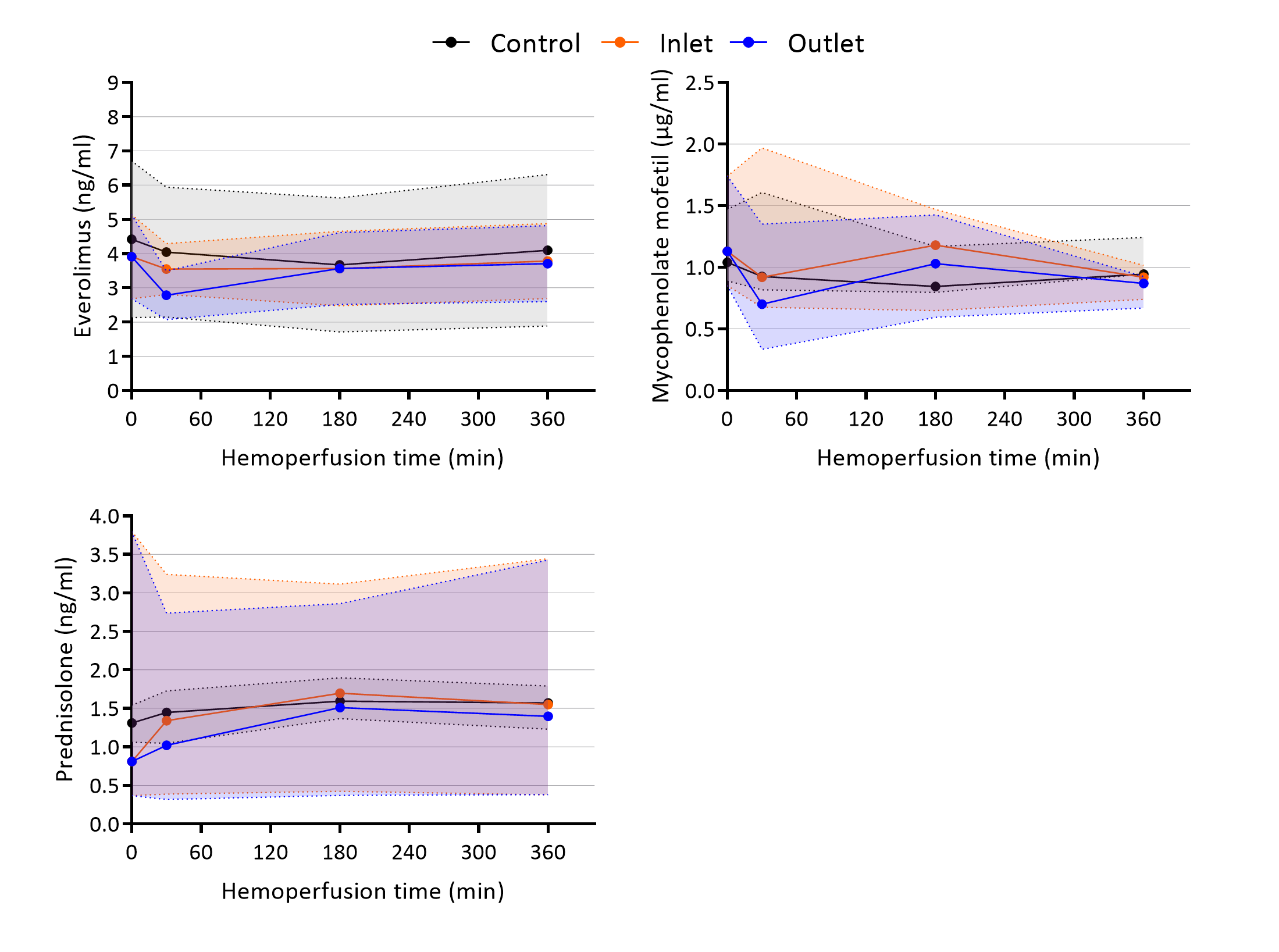


Supplemental Figure 3. Everolimus, Mycophenolate Mofetil and Prednisolone: Drug levels measured in In- and Outlet of CytoSorb® versus controls. Data are displayed as median (line) and interquartile range (shaded area).


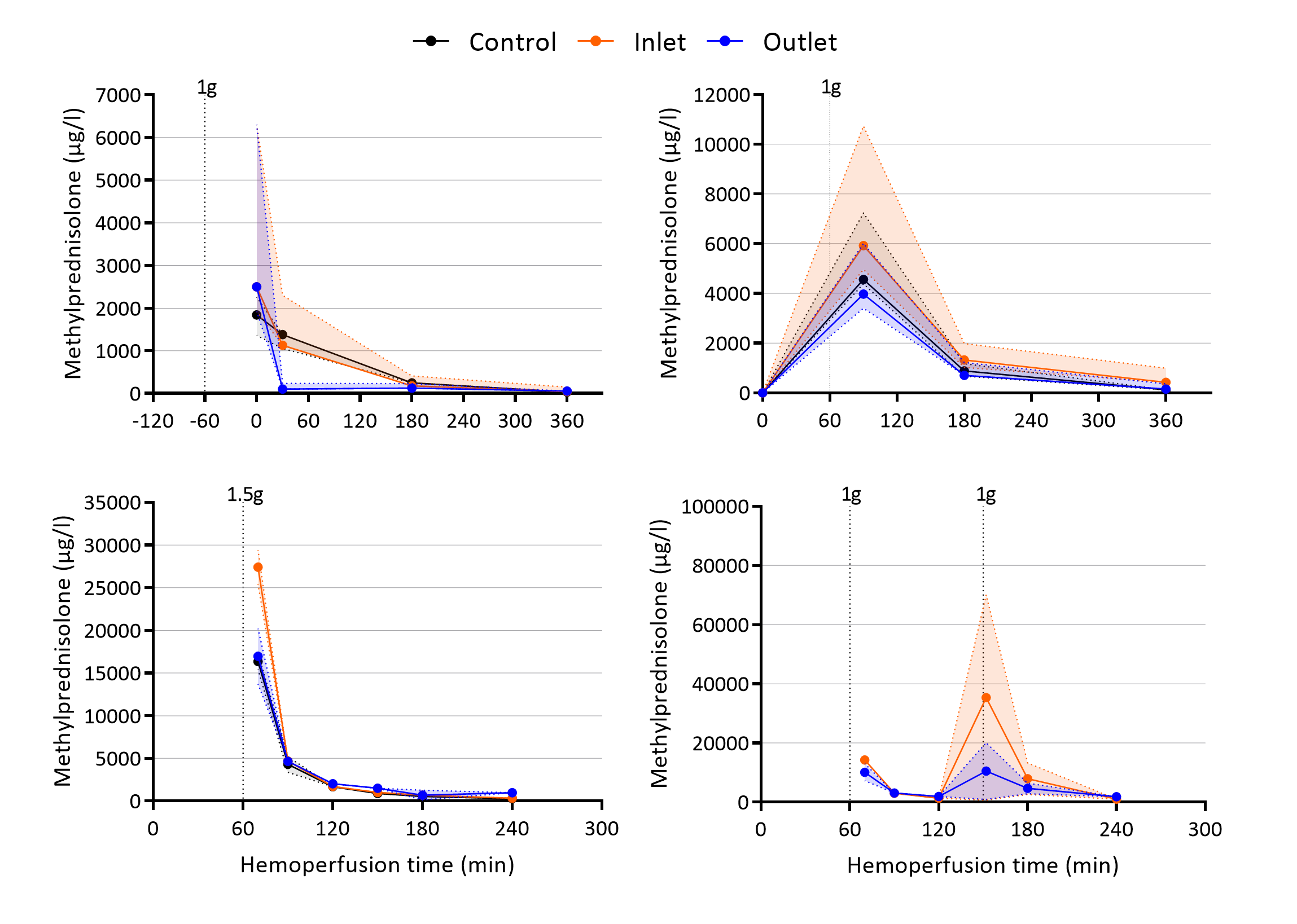


Supplemental Figure 4. Methylprednisolone: Drug levels measured at the In- and Outlet of CytoSorb® versus controls. Data are displayed as median (line) and interquartile range (shaded area).


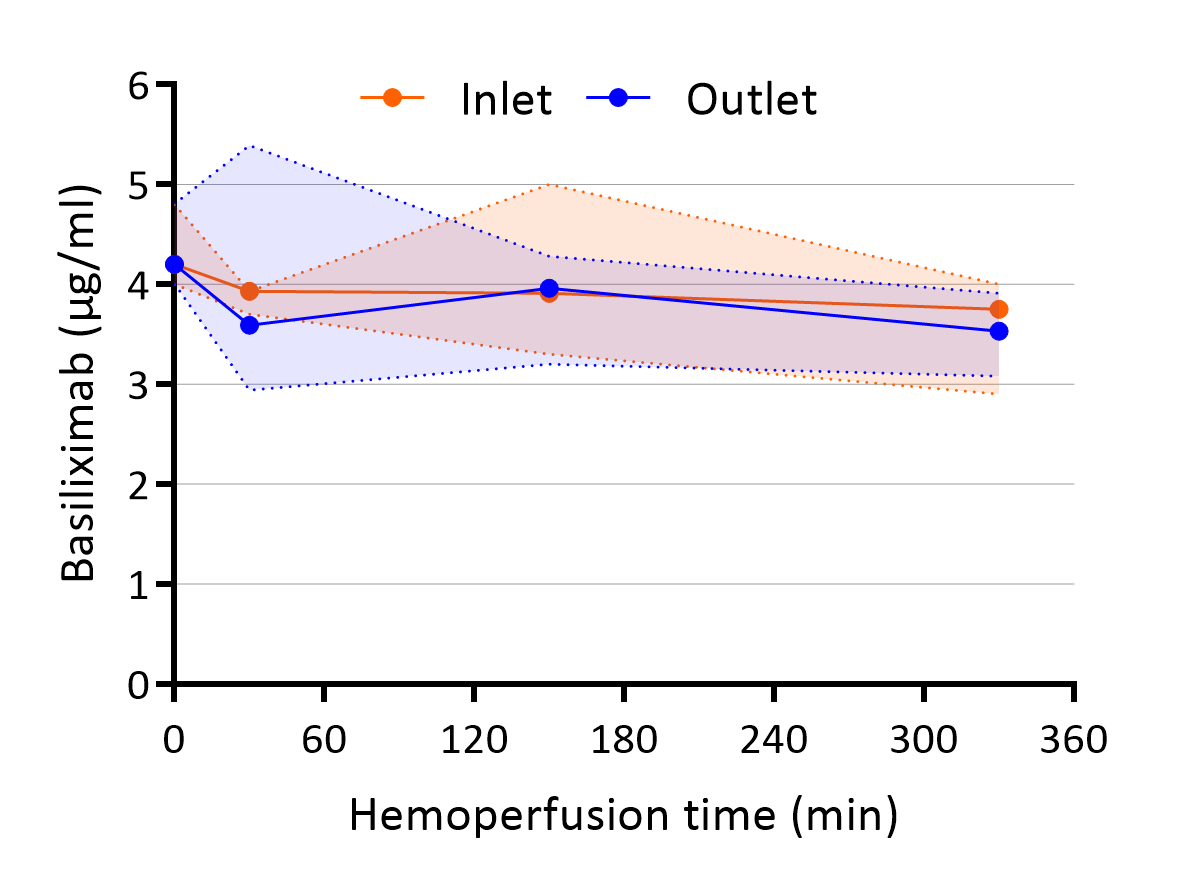


Supplemental Figure 5. Basiliximab: Drug levels measured at the in- and outlet of CytoSorb®. Data are displayed as median (line) and interquartile range (shaded area).


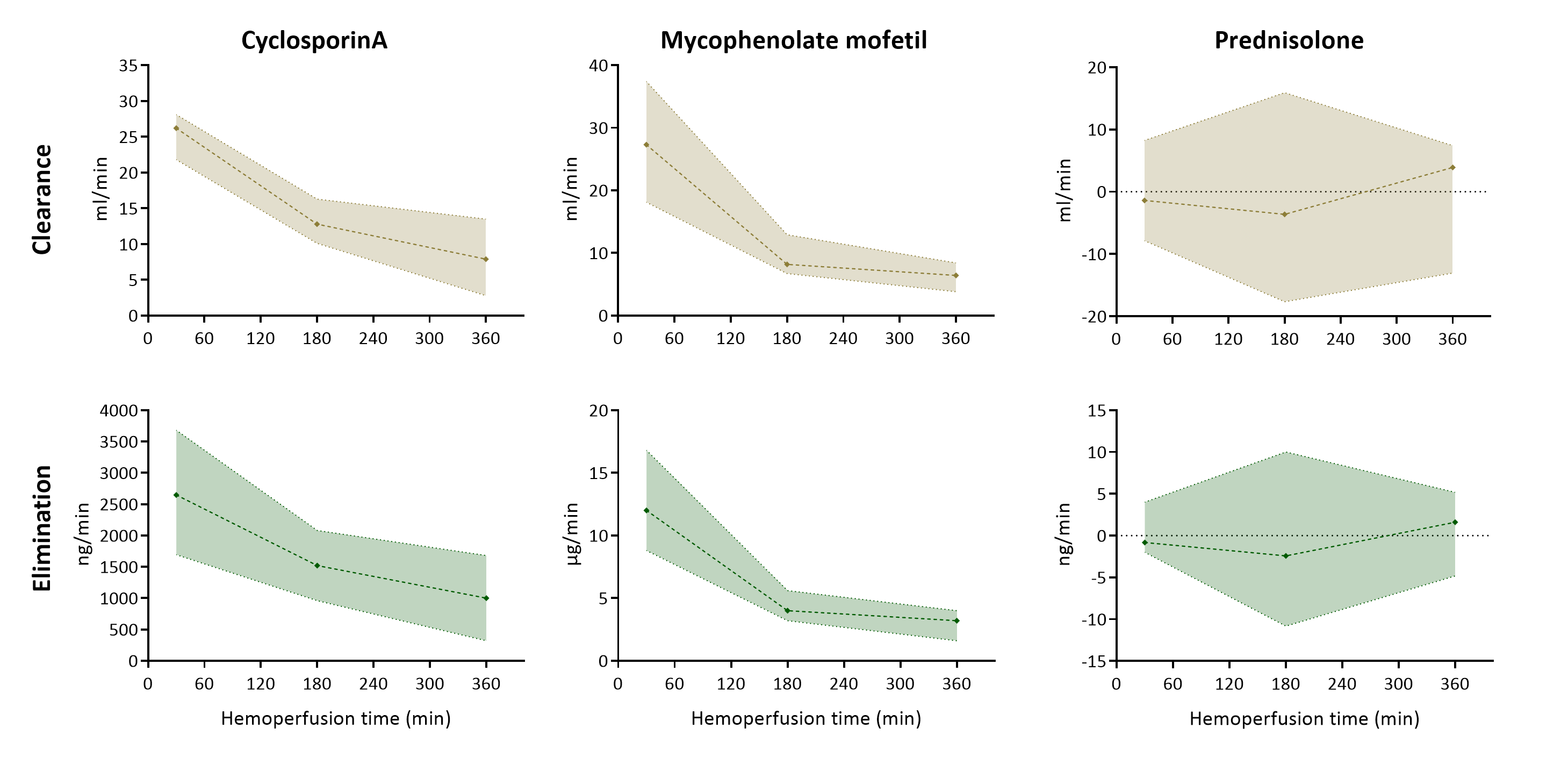

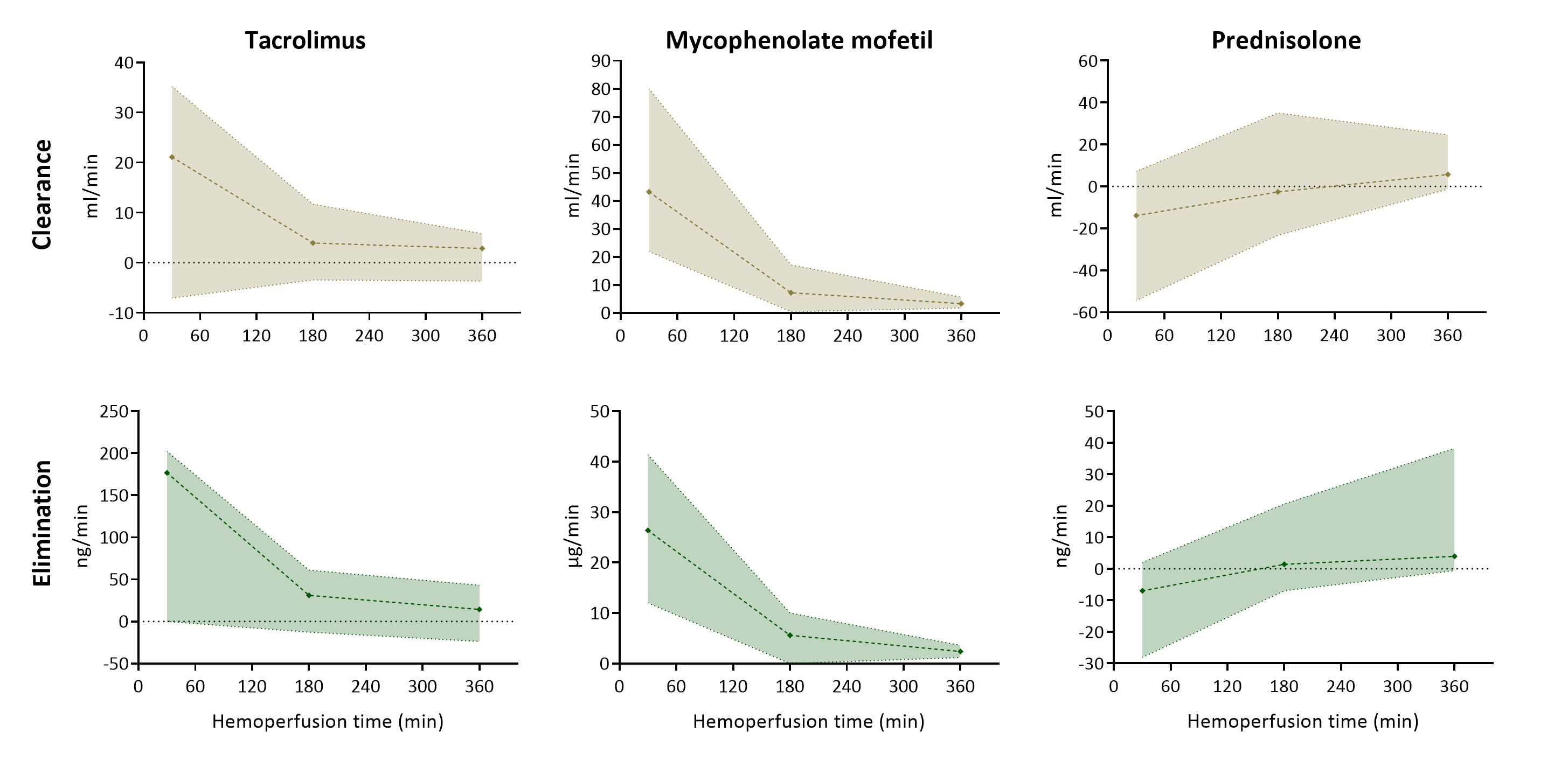

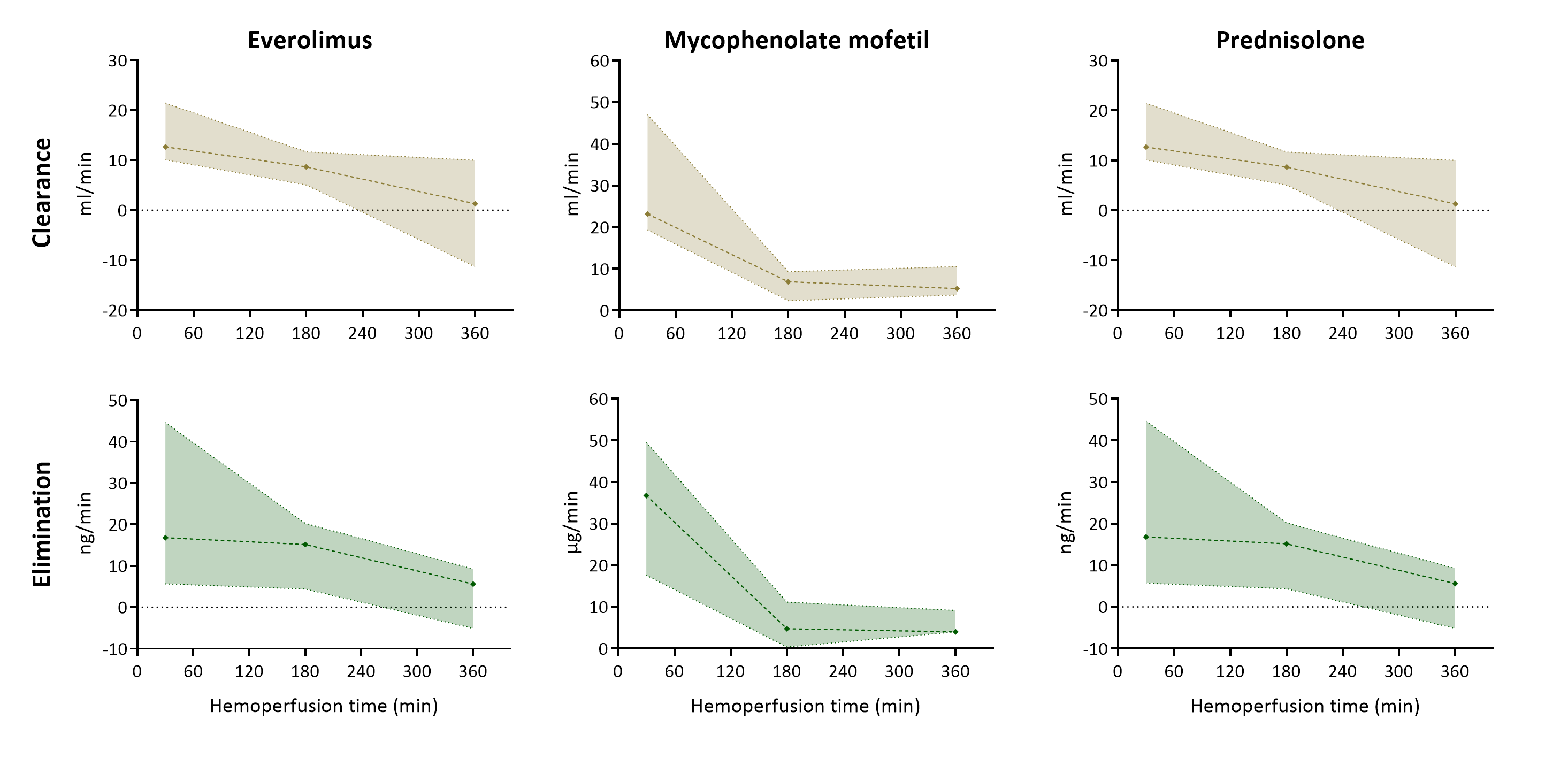


Supplemental Figure 6. Clearance rates (left panels) and elimination rates (right panels) of CYA, MMF, PRE, TAC, EVER. Data are displayed as median (line) and interquartile range (shaded area).


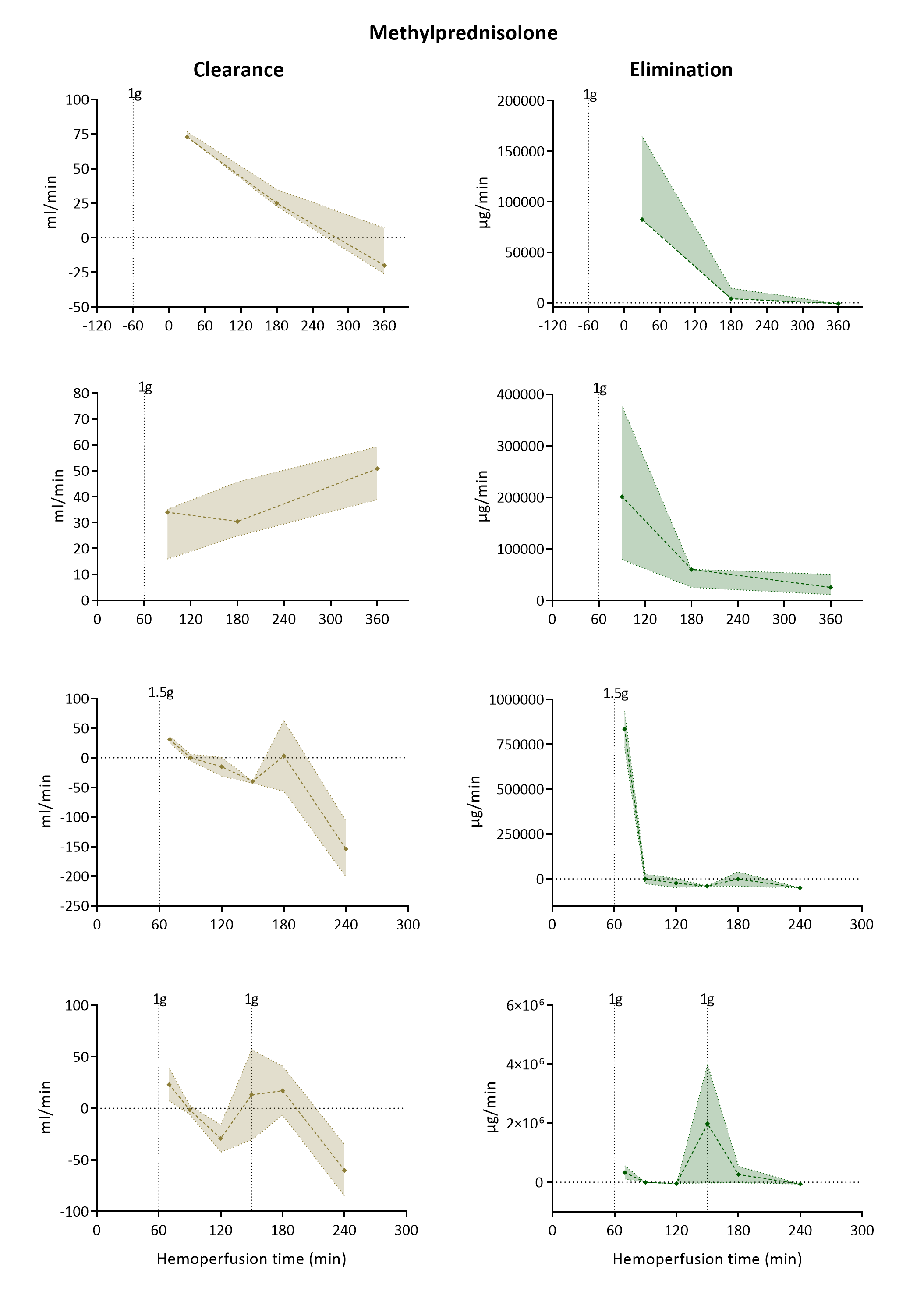


Supplemental Figure 7. Clearance rates (left panels) and elimination rates (right panels) of MP in different experimental scenarios. Data are displayed as median (line) and interquartile range (shaded area).


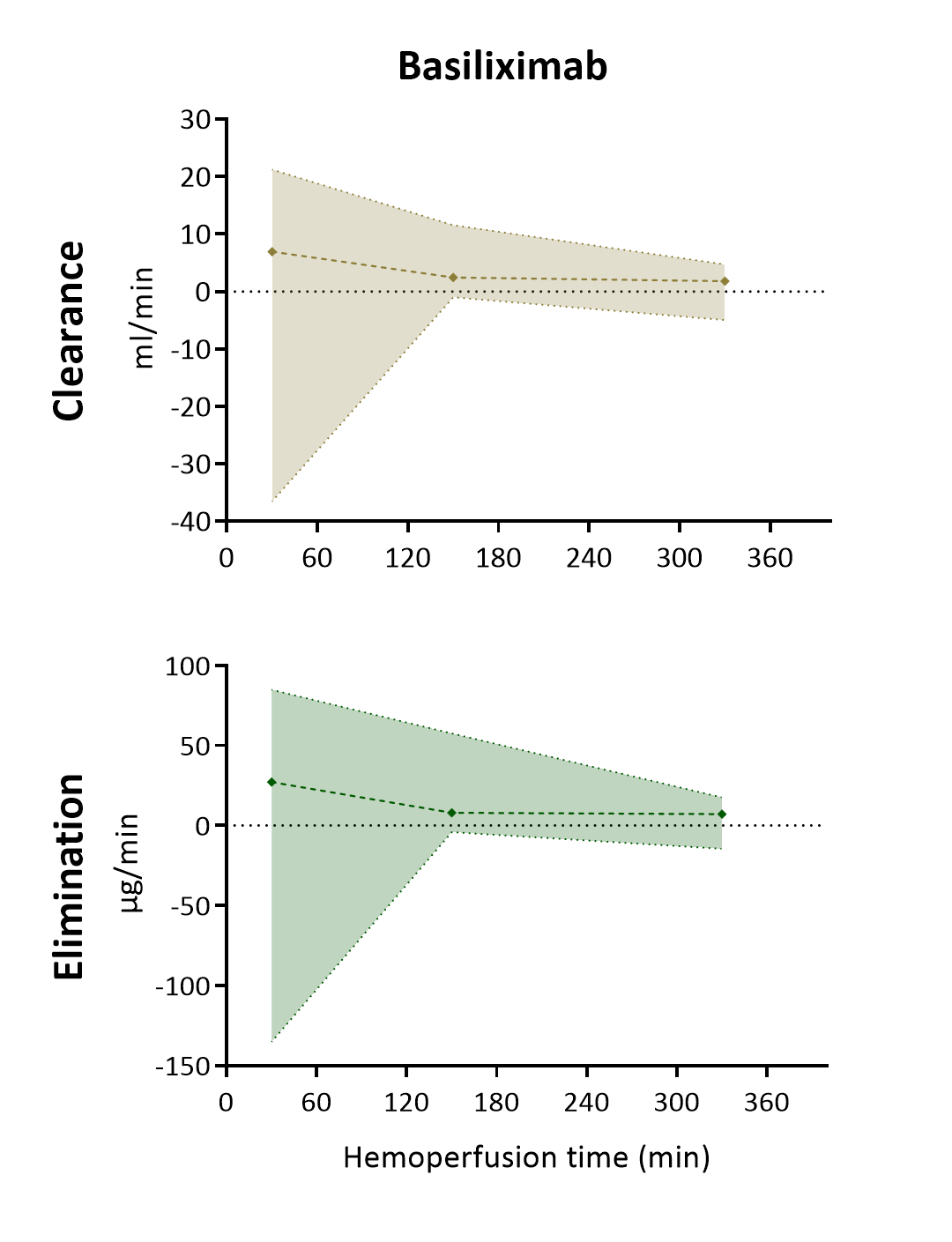


Supplemental Figure 8. Clearance rates (left panels) and elimination rates (right panels) of BAS. Data are displayed as median (line) and interquartile range (shaded area).

Supplemental Figure 9. Goodness-of-fit plots for the final population pharmacokinetic models. Points: observations, lines: line of identity


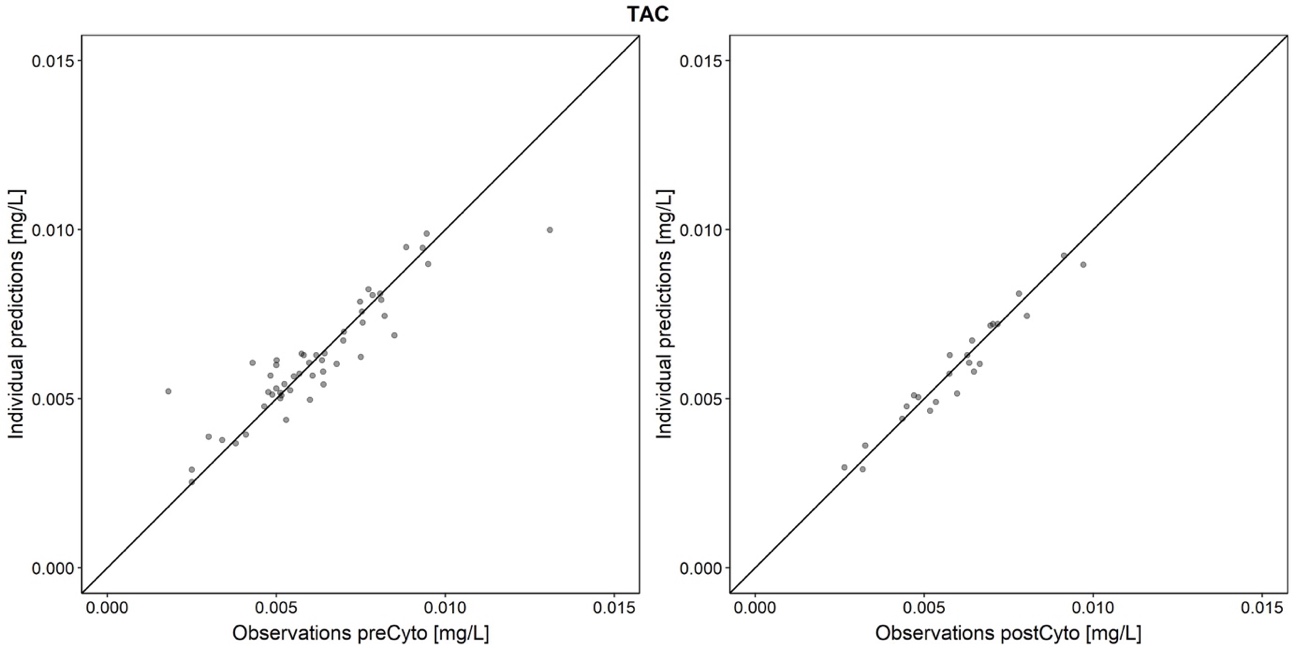

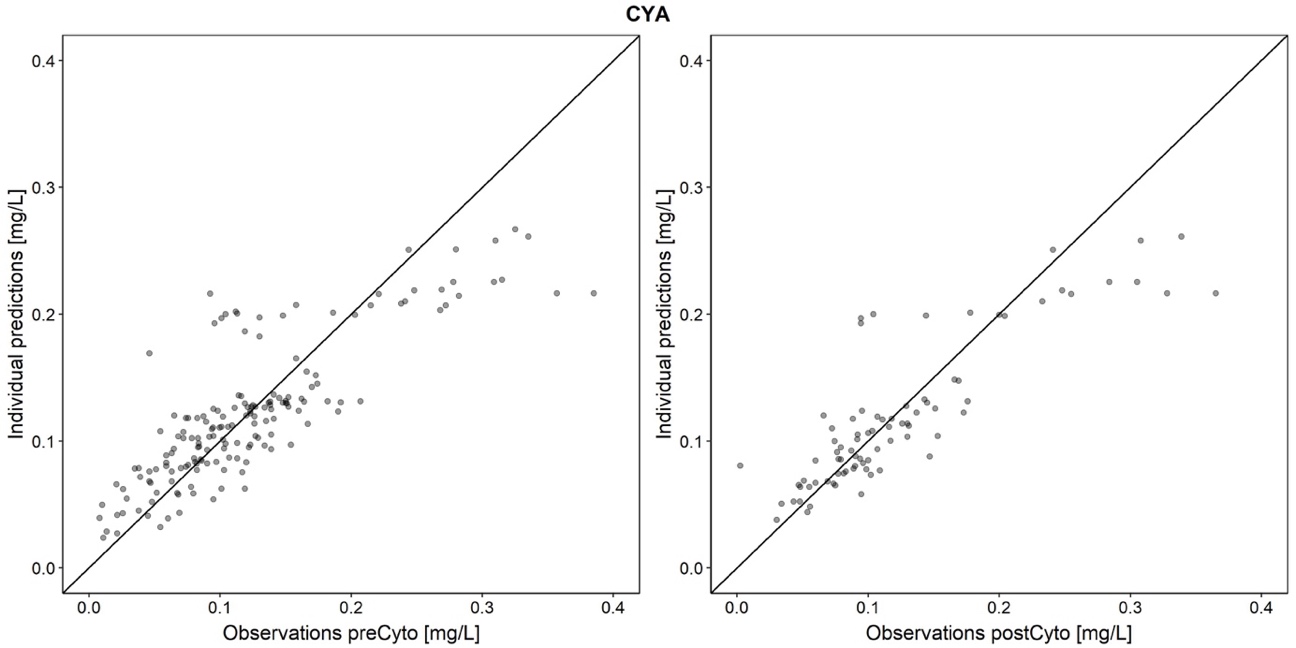

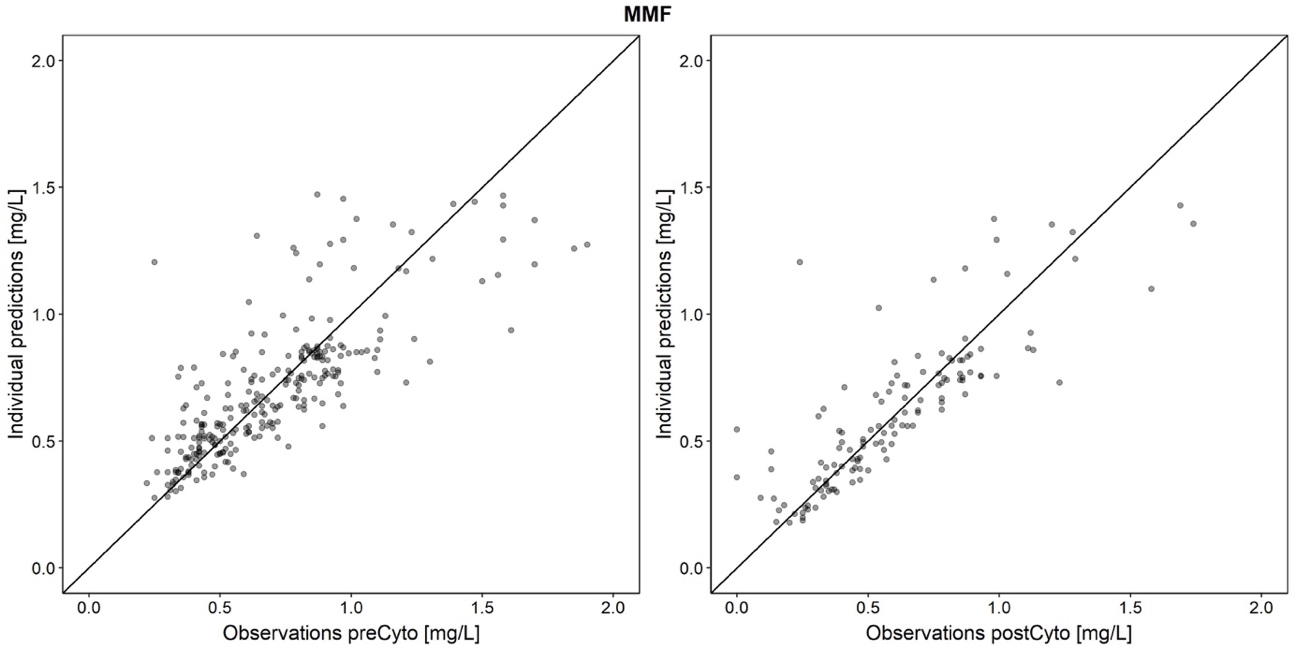


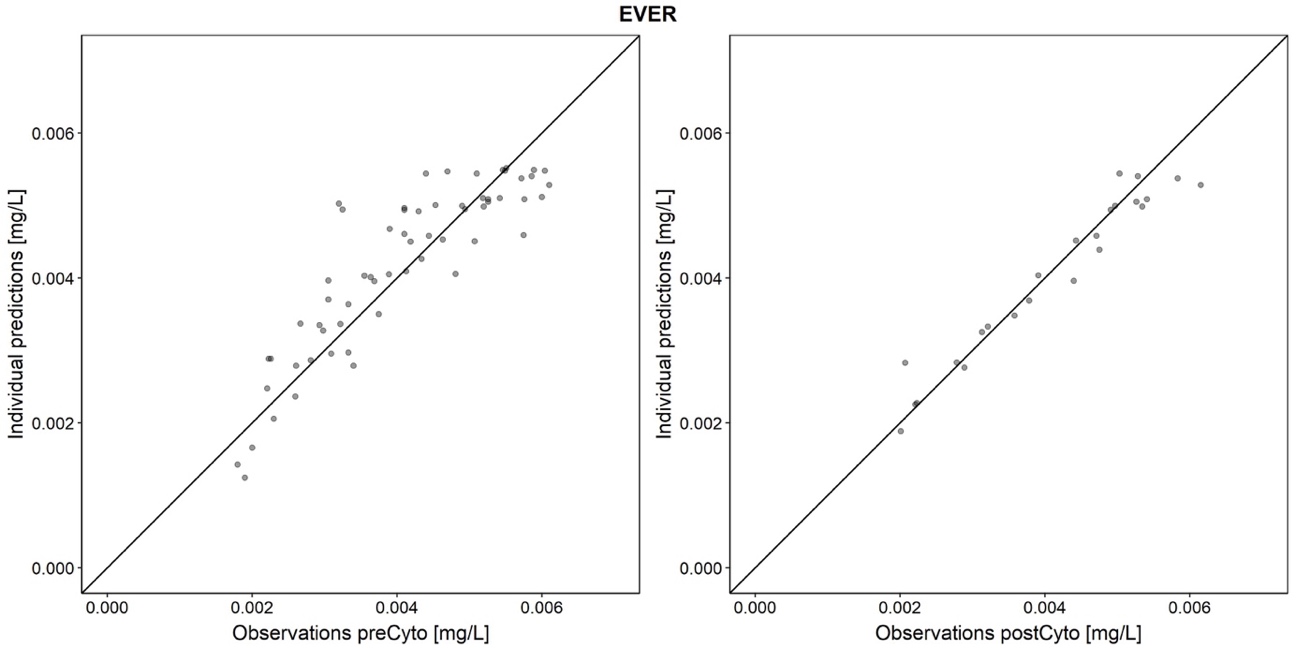


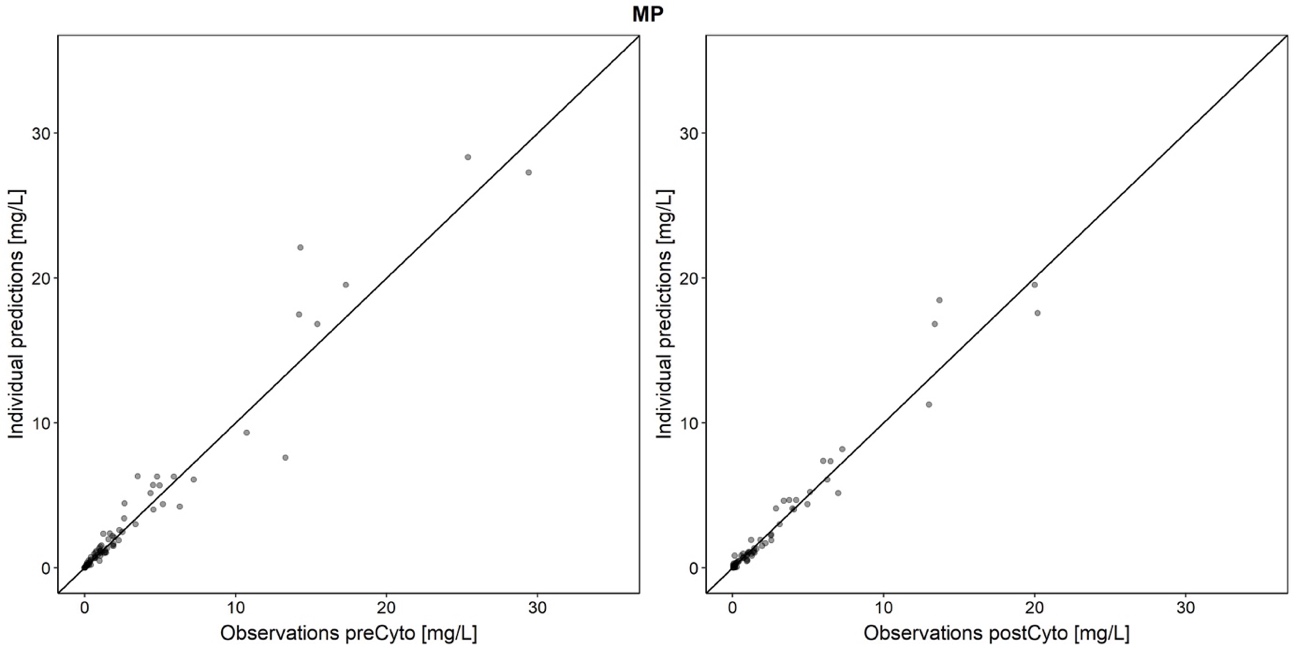


|  | Parameter estimates (RSE) [shrinkage %] | | | | |  |
| --- | --- | --- | --- | --- | --- | --- |
| Parameter [unit] | TAC | CYA | MMF | EVER | MP |  |
| Fixed-effect parameters |  |  |  |  |  |  |
| CL [L/h] |  |  |  |  | 78.9 (9) |  |
| V1 [L] |  |  |  |  | 21.0 (11) |  |
| V2 [L] |  |  |  |  | 34.9 (10) |  |
| Q [L/h] |  |  |  |  | 31.8 (13) |  |
| CL/F [L/h] | 139 (11) | 351 (23) | 112 (6) | 133 (9) |  |  |
| V1/F [L] | 6330 (24) | 826 (48) | 1100 (37) | 3100 (50) |  |  |
| KA [h^-1^] | 4.8 (22) | 0.0076 (48) | 0.090 (27) | 0.232 (40) |  |  |
| ALAG [h] | 15.3 (0) |  | 4.67 (6) | 4.14 (11) |  |  |
| Interindividual variability |  |  |  |  |  |  |
| CL, CV% |  |  |  |  | 28.1 (20) [0] |  |
| CL/F, CV % | 22.9 (23) [8] | 43.8 (19) [0] | 34.2 (11) [2] | 16.4 (21) [0] |  |  |
| V/F, CV % | 41.1 (30) [16] |  |  |  |  |  |
| Residual variability |  |  |  |  |  |  |
| Prop. error, CV % | 16.4 (20) | 34.5 (10) | 25.2 (8) | 16.4 (9) | 30.1 (16) |  |

Supplemental Table 1. Parameter estimates for the structural pharmacokinetic models

Abbreviations: RSE: Relative standard error, CL: Clearance, V1: Central volume of distribution, V2: Peripheral volume of distribution, Q: Intercompartmental clearance, F: Bioavailability, ALAG: Delay of oral adsorption after oral intake, KA: First order oral absorption rate, CV: Coefficient of variation

|  | Parameter estimates (RSE [%]) | | | | |  |
| --- | --- | --- | --- | --- | --- | --- |
| Parameter [unit] | TAC | CYA | MMF | EVER | MP |  |
| Fixed-effect parameters |  |  |  |  |  |  |
| CL _sheep_ [L/h] |  |  |  |  | 75.1 (9) |  |
| CL/F _sheep_ [L/h] | 132 (9) | 343 (11) | 113 (5) | 130 (7) |  |  |
| A_max_ CytoSorb [mg] | 0.040 (16) | 1.15 (34) | 4.17 (27) | 0.0163 (29) | 53.4 (31) |  |
| CL_max_ CytoSorb [L/h] | 4.02 (12) | 2.80 (23) | 3.71 (16) | 3.23 (22) | 8.21 (13) |  |
| Residual variability |  |  |  |  |  |  |
| Prop. error pre, CV % | 15.6 (18) | 33.7 (10) | 25.1 (8) | 15.8 (9) | 32.3 (23) |  |
| Add. error pre [mg] |  |  |  |  | 0.0042 (12) |  |
| Prop. error post, CV % | 8.8 (13) | 29 (13) | 27.8 (11) | 9.0 (25) | 26.2 (20) |  |
| Add. error post [mg] |  |  |  |  | 0.129 (81) |  |

Supplemental Table 2. Parameter estimates for the adsorption pharmacokinetic models.

Abbreviations: RSE: Relative standard error, CL: Clearance, F: bioavailability, CV: Coefficient of variation, A_max_: maximum drug amount that can be adsorbed, A_half_: drug amount associated with half of the maximum capacity, CL_max_: maximum clearance
